# Hypertension and risk of prostate cancer: a systematic review and meta-analysis

**DOI:** 10.1038/srep31358

**Published:** 2016-08-11

**Authors:** Zhen Liang, Bo Xie, Jiangfeng Li, Xiao Wang, Song Wang, Shuai Meng, Alin Ji, Yi Zhu, Xin Xu, Xiangyi Zheng, Liping Xie

**Affiliations:** 1 Department of Urology, First Affiliated Hospital, School of Medicine, Zhejiang University, Hangzhou, 310003, China; 2 Department of Urology, Tongde Hospital of Zhejiang Province, Hangzhou, Zhejiang 310012, China; 3 Department of Urology, Zhejiang Provincial People’s Hospital, Hangzhou, Zhejiang 310014, China

## Abstract

The previously reported association between hypertension and prostate cancer risk was controversial. We performed this systematic review and meta-analysis of all available studies to summarize evidence on this association. Studies were identified by searching PubMed, Web of Science and Chinese National Knowledge Infrastructure (CNKI) databases through January 2016. Pooled relative risks (RRs) with their corresponding 95% confidence intervals (CIs) were calculated using a random-effects model. A total of 21 published studies were included in this meta-analysis. A significant increase in the risk of prostate cancer (RR 1.08, 95% CI 1.02–1.15, *P* = 0.014) was observed among individuals with hypertension. There was statistically significant heterogeneity among included studies (*P* < 0.001 for heterogeneity, *I*^*2*^ = 72.1%). No obvious evidence of significant publication bias was detected by either Begg’s test (*P* = 0.174) or Egger’s test (*P* = 0.277). In conclusion, this meta-analysis indicates that hypertension may be associated with an increased risk of prostate cancer. Considering the substantial heterogeneity and residual confounding among included studies, further large-scale, well-designed prospective cohorts, as well as mechanistic studies, are urgently needed to confirm our preliminary findings.

Prostate cancer has the second highest incidence of all cancers in males worldwide, with 1,111,700 new cases and 307,500 deaths estimated to have occurred in 2012 1. Incidence of prostate cancer varies across different geographic regions, with a greater prevalence in Western countries (i.e., the United States and Western Europe) than in Asian countries (i.e., China and Japan)^2^. However, in the past decade, the largest increase in incidence was seen for cancer of the prostate in China^3^. Age, race/ethnicity, and family history of prostate cancer are the most definitive risk factors for prostate cancer^4^. Unhealthy behaviors (i.e., lack of physical activity^5^) and eating too few vegetables (i.e., carrots^6^ and cruciferous vegetables^7^) also have been reported to be associated with prostate cancer risk, although controversies still exist.

Recently, several researchers have explored whether hypertension is a potential risk factor for prostate cancer with conflicting results. Two case–control studies and two cohort studies^8^,^9^,^10^,^11^ suggested that individuals with hypertension had an increased risk of prostate cancer compared with subjects without hypertension. In contrast, several other studies^12^,^13^,^14^ failed to demonstrate a positive correlation between hypertension and prostate cancer risk. Stocks *et al*.^15^ even reported a significantly negative association between them. Given the conflicting results as discussed above, we conducted this meta-analysis to summarize evidence on the relationship between hypertension and the risk of prostate cancer.

## Results

### Literature search and study characteristics

**Figure 1:**
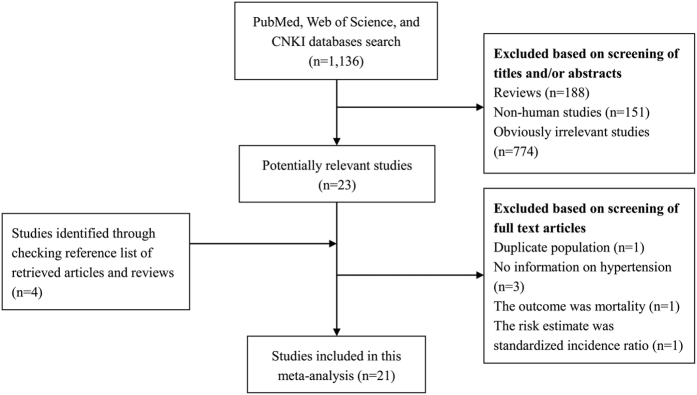
Process of literature search and study selection.

The detailed process of literature search is presented in Fig. 1. 21 published studies^8^,^9^,^10^,^11^,^12^,^13^,^14^,^15^,^16^,^17^,^18^,^19^,^20^,^21^,^22^,^23^,^24^,^25^,^26^,^27^,^28^ were eventually included in this meta-analysis of the association between hypertension and prostate cancer risk. These studies (14 cohort, 3 nested case-control and 4 case-control studies) were carried out in the following geographical regions: Europe (n = 9), America (n = 8), and Asia (n = 4). All of the included studies were published between 1997 and 2015, including a total of 24,366 cases. Information on exposure (hypertension) and outcome (prostate cancer) was mainly gained from physical examination and cancer registry, respectively. The scores of study quality, evaluated by the Newcastle-Ottawa Scale (NOS), ranged from 4 to 8 (with a mean of 6.1). Table 1 lists the general characteristics of all studies included in the present meta-analysis.

### Overall analysis and evaluation of heterogeneity

**Figure 2:**
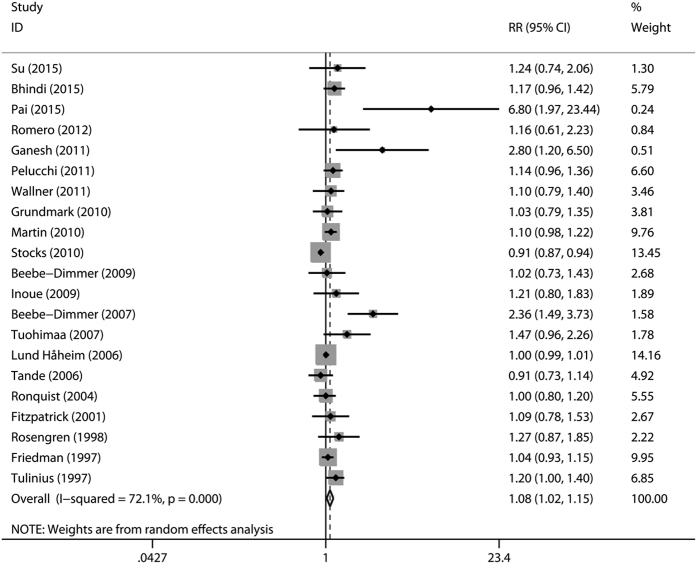


The multivariable-adjusted relative risks (RRs) for each individual study and for the combination of all included studies are presented in Fig. 2. A significant increase in the risk of prostate cancer (RR 1.08, 95% confidence interval (CI) 1.02–1.15, *P* = 0.014) was observed among individuals with hypertension. There was statistically significant heterogeneity among included studies (*P* < 0.001 for heterogeneity, *I*^*2*^ = 72.1%).

### Subgroup analysis

Next, we performed stratified analyses by geographical region, study design, study quality, and number of cases (Table 2). In the subgroup analysis based on geographical region, more pronounced association was detected in studies from Asia (RR 1.88, 95% CI 1.04–3.38) compared with studies from Europe (RR 1.04, 95% CI 0.97–1.11) and America (RR 1.11, 95% CI 0.97–1.27). When further stratified by study design, the RRs (95% CI) were 1.05 (0.99-1.11) and 1.49 (1.00–2.22) for cohort/nested case-control and case-control studies, respectively. Finally, in the stratified analyses by study quality and number of included cases, statistically significant associations were observed in those studies with high quality (RR 1.16, 95% CI 1.01–1.33) and small sample size (RR 1.15, 95% CI 1.03–1.29) rather than in studies with low quality (RR 1.06, 95% CI 0.98–1.15) or large sample size (RR 1.06, 95% CI 0.95–1.20).

### Sensitivity analysis

**Figure 3:**
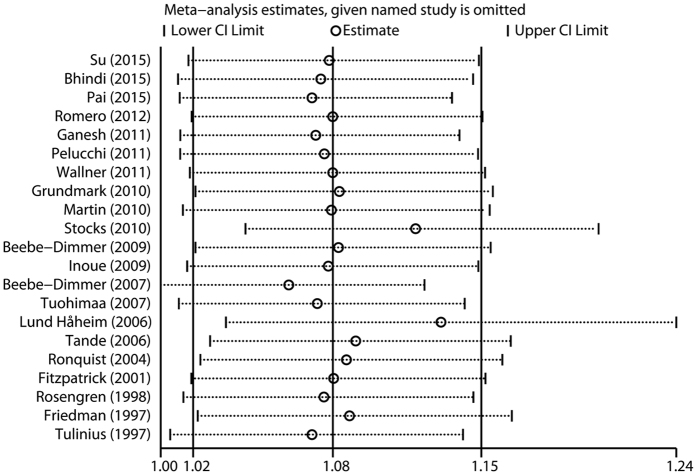
Sensitivity analysis was conducted by excluding each study in turn and recalculating the combined risk estimates.

The influence of each study on the pooled RR was evaluated by repeating the overall analysis after omitting each study in turn. The results indicated that no single study dominated the combined RR. The 21 study-specific RRs ranged from a low of 1.06 (95% CI 1.00–1.12) to a high of 1.13 (95% CI 1.03–1.24) via omission of the study by Beebe-Dimmer *et al*.^10^ and the study by Lund Håheim *et al*.^24^, respectively (Fig. 3).

### Cumulative meta-analysis

**Figure 4:**
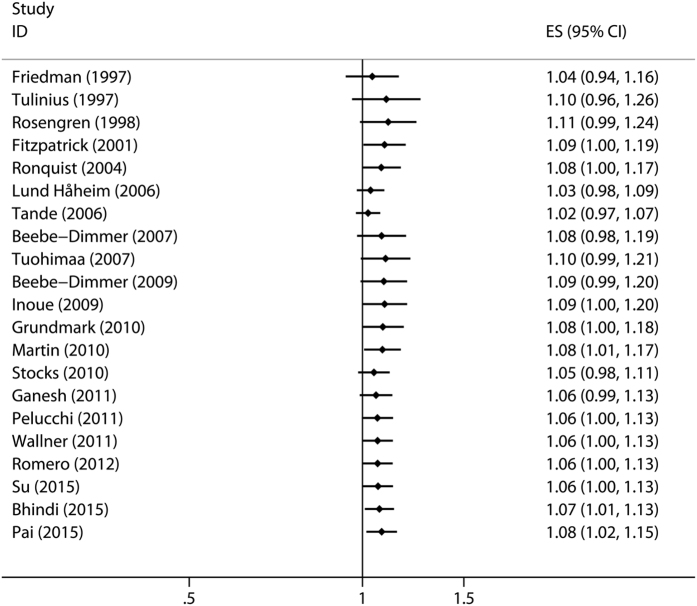
Cumulative meta-analysis was conducted according to publication year.

Cumulative meta-analysis is the process of repeated pooling of individual studies each time adding a new study. In this meta-analysis, we carried out the cumulative meta-analysis according to publication year. As shown in Fig. 4, the combined RR achieved statistical significance when the study by Bhindi *et al*.^17^ completed in 2015 was added.

### Publication bias

There was no obvious evidence of significant publication bias by Begg’s test (*P* = 0.174) or Egger’s test (*P* = 0.277).

## Discussion

This systematic review and meta-analysis summarized the findings of observational studies on the association between hypertension and prostate cancer risk, including 17 cohort/nested case-control studies and 4 case-control studies. The results indicated that individuals with hypertension had a significant increased risk of prostate cancer.

The findings of this meta-analysis were in agreement with a previous meta-analysis^29^, which indicated that hypertension was associated with a significant 15% (*P* = 0.035) greater risk of prostate cancer. However, that pooled analysis only included 10 published studies, with a total of 4,343 cases. By contrast, the present meta-analysis included more recent studies and thereby had larger sample size (a total of 24,366 cases); potentially improved statistical power.

The exact mechanism underlying the positive association between hypertension and prostate cancer risk is not clear. It has been proposed that hypertension could increase the risk of prostate cancer through the activity of the sympathetic nervous system that can lead to androgen-mediated stimulation of prostate cancer cell growth^18^. In studies with hypertensive animal models, hypertension can result in abnormal proliferation and a defective growth stimulatory-inhibitory control^30^. On the other hand, individuals using renin-angiotensin system (RAS) inhibitors, an antihypertensive drug, have been reported to be associated with a reduced risk of prostate cancer (RR 0.92, 95% CI 0.87–0.98)^31^.

Our study had several strengths. A total of 21 published studies with 24,366 prostate cancer cases were pooled in this meta-analysis, which might enhance the statistical power of the data analysis and thus provide more reliable estimates. Various stratified analyses and sensitivity analyses were performed to explore the sources of heterogeneity and assess the robustness of the combined risk estimate. The estimates for the most fully adjusted model reported in each study were extracted in this study to reduce the potential confounding effect.

There were also several important limitations that need to be considered in interpreting the results of this study. First, substantial heterogeneity was observed across individual studies (*P* < 0.001 for heterogeneity, *I*^*2*^ = 72.1%), which might distort the combined estimates. Heterogeneity is caused by variation in definitions and ranges of exposure, methods of exposure and outcome assessment, and population sources. Heterogeneity could also be attributed to the heterogeneity of the prostate cancer disease and the divergent results between hypertension and prostate cancer diagnosis and death^15^. Second, although Begg’s and Egger’ test did not show any evidence of publication bias, some inevitable publication bias might exist. Small negative studies were less likely to be published and gray literature, due to its diverse origins and unpublished nature, may be difficult to find. Third, a meta-analysis is unable to solve problems with confounding factors that could be inherent in the original studies. Although the majority of included studies adjusted for a wide range of potential confounders for prostate cancer, residual or unknown confounding variables cannot be completely excluded as a potential interpretation for the findings of current meta-analysis. Inadequate control of all known confounding variables may bias the pooled risk estimate, toward exaggeration or underestimation of effect size. Fourth, the cutoff points for the high and low blood pressure groups were various in included studies, which might contribute to the heterogeneity and have an influence on the summary risk estimate. Fifth, this study lacked the data of tumor characteristics. Prostate cancer is a heterogeneous disease and thus prostate cancer patients have very different characteristics, progression and survival. Lastly, subjects with hypertension are under increased medical monitoring, which can cause detection bias, especially after PSA screening was introduced in the early 1990s. A large proportion of PSA-detected cancers have been low-risk tumors, which may dilute an association between metabolic factors and high risk tumors^32^.

## Conclusion

This meta-analysis indicates that hypertension may be associated with an increased risk of prostate cancer. Considering the substantial heterogeneity and residual confounding among included studies, further large-scale, well-designed prospective cohorts, as well as mechanistic studies, are urgently needed to confirm our preliminary findings.

## Materials and Methods

### Literature search

A comprehensive literature search of published studies was performed in January 2016 based on PubMed, Web of Science, and the Chinese National Knowledge Infrastructure (CNKI) databases with the following search algorithm: (“hypertension” or “blood pressure” or “systolic pressure” or “diastolic pressure”) and (“prostate cancer” or “prostate neoplasm”) and (“cohort” or “case control” or “case-control”). In addition, the lists of references from retrieved articles and reviews were also checked to identify any additional eligible studies. No limitations on language or publication date were applied. This systematic review and meta-analysis was designed, performed, and reported based on the standards of quality for reporting meta-analyses^33^.

### Study selection

A study was included in this meta-analysis if it met all of the following criteria: (*i*) the exposure of interest was hypertension; Hypertension is defined as a systolic blood pressure above 140 mmHg or a diastolic blood pressure above 90 mmHg. (*ii*) the outcome of interest was prostate cancer; (*iii*) study design was cohort, nested case-control or case-control; and (*iv*) the risk estimates with their corresponding 95% CIs were available (or enough data were provided to estimate effect size). If multiple publications reported data from the same study, the publication with the largest sample size was included in the meta-analysis.

### Quality assessment

Two investigators (Z.L. and X.X.) assessed the quality of each study independently by using the NOS (http://www.ohri.ca/programs/clinical_epidemiology/oxford.asp). NOS is an eight-item instrument designed to assess selection (four items), comparability (one item), and exposure/outcome (three items). Each item represents one point, except for comparability (two points). Thus the range of potential scores is 0–9 points. A study is classified as high quality if the score is 7–9 points.

### Data extraction

The following information were gained from each study: first author’s name, publication date, geographical region, study design, age, number of cases, method of exposure and outcome assessment, adjusted risk estimates with their corresponding 95% CIs, and matched or adjusted variables in the design or statistical analysis. Information was collected independently by two authors (Z.L. and X.X.) and any discrepancies were subject to discussion.

### Statistical methods

According to rare disease assumption, the OR (odds ratio) was assumed approximately the same as RR, and the RR was designated as the study outcome. RRs and their 95% CIs were used to calculate and assess the strength of the relationship between hypertension and prostate cancer risk. A random-effects model reported by DerSimonian and Laird^34^, which consider both between-study and within-study variability, was applied to estimate the pooled RR and its 95% CI. Stratified analyses were conducted based on geographical region, study design, study quality, and number of cases.

Cochran *Q* and the *I*^*2*^ index^35^ were adopted to evaluate the heterogeneity of RRs among the included studies. The level of significancefor Cochrane *Q* was set to 0.1 (10%). The value of *I*^*2*^ was used to describe the degree of heterogeneity (*I*^*2*^ < 25%: no heterogeneity; *I*^*2*^ = 25–50%: moderate heterogeneity; *I*^*2*^ > 50%: large or extreme heterogeneity).

Sensitivity analysis was conducted by omitting each study in turn and recalculating the combined RR to determine the influence of each study on the overall risk estimate. Cumulative meta-analysis was also performed by sorting the studies according to publication date.

Begg’s test (rank correlation method)^36^ and Egger’s test (linear regression method)^37^ were applied to evaluate the potential publication bias. All of the statistical analyses were completed using STATA 11.0 (StataCorp, College Station, TX), using two-sided *P* values (set at 0.05).

## Additional Information

**How to cite this article**: Liang, Z. *et al*. Hypertension and risk of prostate cancer: a systematic review and meta-analysis. *Sci. Rep.* **6**, 31358; doi: 10.1038/srep31358 (2016).

## Figures and Tables

**Table 1:** Characteristics of the studies included in meta-analysis of association between hypertension status and prostate cancer risk.

Study	Year	Region	Study design	No. of cases	Age (yr)	Exposure assessment	Outcome assessment	Matched or adjusted factors	NOS score
Su *et al*.	2015	Taiwan	Nested case-control	74	54.87 (SD 18.69)	Database	Database	Age, sex, residence, and insurance premium	7
Bhindi *et al*.	2015	Canada	Cohort	2,235	64.9	Measurement	Biopsy	Age, ethnicity, family history of prostate cancer, prostate volume, history of any prior biopsy, and 5a-reductase inhibitor use	8
Pai *et al*.	2015	Taiwan	Cohort	1,971	69.31 (SD 9.31)	Database	Database	Age, income, urbanization level, and index day	7
Romero *et al*.	2012	Brazil	Cohort	58	≥40	Measurement	Biopsy	Age	5
Ganesh *et al*.	2011	India	Case-control	123	64	Questionnaire	Histologically proven	Age, religion, and education	4
Pelucchi *et al*.	2011	Italy	Case-control	1,294	66 (46–74)	Self-reported	Histologically confirmed	Age, study center, education, smoking, alcohol, physical activity, family history of prostate cancer, and nonalcoholic energy intake	7
Wallner *et al*.	2011	US	Cohort	206	40–79	Physician-diagnosed	Biopsy	Age	6
Grundmark *et al*.	2010	Sweden	Cohort	237	NA	Measurement	Database	NA	5
Martin *et al*.	2010	Norway	Cohort	1,974	50.3 (SD 15.2)	Physical examination	Cancer registry	Age, height, BMI, smoking, marital status, education, physical activity, diabetes, and country of origin	8
Stocks *et al*.	2010	Sweden	Cohort	10,002	70.1 (SD 8.4)	Health examination	Cancer registry	Age, smoking, and BMI	7
Beebe-Dimmer *et al*.	2009	US	Case-control	637	62	Medical records	Database	Age, PSA screening, diabetes, BMI, HDL, and triglycerides	6
Inoue *et al*.	2009	Japan	Cohort	86	40–69	Measurement	Cancer registry	Age, study area, smoking, ethanol intake, and serum cholesterol	7
Beebe-Dimmer *et al*.	2007	US	Case-control	139	40–79	Questionnaire	Cancer registry	Age and smoking history	7
Tuohimaa *et al*.	2007	Finland	Nested case-control	132	62.1 (SD 4.9)	Measurement	Cancer registry	Vitamin D	5
Lund Håheim *et al*.	2006	Norway	Cohort	507	40–49	Questionnaire	Cancer registry	Age	5
Tande *et al*.	2006	US	Cohort	385	45–64	Clinical examination	Cancer registry	Age, race, family history, education, smoking, ethanol intake, caloric intake, and milk intake	6
Ronquist *et al*.	2004	UK	Nested case-control	1,013	50–79	Database	Medical record	Age, smoking, BMI, alcohol, diabetes, IHD, HF, prostatism and calendar year	6
Fitzpatrick *et al*.	2001	US	Cohort	209	≥65	Clinical examination	Medical record	Age, race, and BMI	6
Rosengren *et al*.	1998	Sweden	Cohort	263	47–55	Screening examination	Cancer registry	Age	5
Friedman *et al*.	1997	US	Cohort	2,297	30–79	Health checkup	Cancer registry	Age, race, BMI, and alcohol consumption	7
Tulinius *et al*.	1997	Iceland	Cohort	524	50.3 (SD 11)	Questionnaire	Cancer registry	Age	5

PSA, prostate-specific antigen; NOS, Newcastle-Ottawa Scale; yr, year; SD, standard deviation; BMI, body mass index; IHD, ischemic heart disease; HF, heart failure; HDL, high density lipoprotein; NA, not available.

**Table 2:** Subgroup analyses of the association between hypertension and prostate cancer risk.

Subgroup	Included studies	No. of cases	Pooled RR (95% CI)	*P*	Heterogeneity		
					Q	I^2^ (%)	*P*
Total	21		1.08 (1.02–1.15)	0.014	71.70	72.1	<0.001
Study design							
Cohort/nested case-control	17	22,173	1.05 (0.99–1.11)	0.115	50.30	68.2	<0.001
Case-control	4	2,193	1.49 (1.00–2.22)	0.050	13.31	77.5	0.004
Geographical region							
America	8	6,166	1.11 (0.97–1.27)	0.121	14.70	52.4	0.040
Europe	9	15,946	1.04 (0.97–1.11)	0.245	37.13	78.5	<0.001
Asia	4	2,254	1.88 (1.04–3.38)	0.036	9.38	68.0	0.025
Study quality							
High (NOS ≥ 7)	9	20,072	1.16 (1.01–1.33)	0.039	49.64	83.9	<0.001
Low (NOS <7)	12	4,294	1.06 (0.98–1.15)	0.124	16.25	32.3	0.132
No. of cases							
≥1000	7	20,786	1.06 (0.95–1.20)	0.303	32.81	81.7	<0.001
<1000	14	3,580	1.15 (1.03–1.29)	0.014	31.03	58.1	0.003

No., number; RR, relative risk; CI, confidence interval; NOS, Newcastle-Ottawa Scale.

## References

[b1] Torre L. A. . Global cancer statistics, 2012. CA Cancer J Clin. 65, 87–108 (2015). 25651787 10.3322/caac.21262

[b2] Marugame T. & Katanoda K. International comparisons of cumulative risk of breast and prostate cancer, from cancer incidence in five continents Vol. VIII. Jpn J Clin Oncol. 36, 399–400 (2006). 16818481 10.1093/jjco/hyl049

[b3] Chen W. . Cancer statistics in China, 2015. CA Cancer J Clin. 66, 115–132 (2016). 26808342 10.3322/caac.21338

[b4] Attard G. . Prostate cancer. Lancet (2015).

[b5] Liu Y. . Does physical activity reduce the risk of prostate cancer? A systematic review and meta-analysis. Eur Urol. 60, 1029–1044 (2011). 21802197 10.1016/j.eururo.2011.07.007

[b6] Xu X. . Dietary carrot consumption and the risk of prostate cancer. Eur J Nutr. 53, 1615–1623 (2014). 24519559 10.1007/s00394-014-0667-2

[b7] Liu B., Mao Q., Cao M. & Xie L. Cruciferous vegetables intake and risk of prostate cancer: a meta-analysis. Int J Urol. 19, 134–141 (2012). 22121852 10.1111/j.1442-2042.2011.02906.x

[b8] Pai P. Y. . Long term antihypertensive drug use and prostate cancer risk: A 9-year population-based cohort analysis. Int J Cardiol. 193, 1–7 (2015). 26002406 10.1016/j.ijcard.2015.05.042

[b9] Ganesh B., Saoba S. L., Sarade M. N. & Pinjari S. V. Risk factors for prostate cancer: An hospital-based case-control study from Mumbai, India. Indian J Urol. 27, 345–350 (2011). 22022057 10.4103/0970-1591.85438 PMC3193734

[b10] Beebe-Dimmer J. L., Dunn R. L., Sarma A. V., Montie J. E. & Cooney K. A. Features of the metabolic syndrome and prostate cancer in African-American men. Cancer 109, 875–881 (2007). 17265528 10.1002/cncr.22461

[b11] Tulinius H., Sigfusson N., Sigvaldason H., Bjarnadottir K. & Tryggvadottir L. Risk factors for malignant diseases: a cohort study on a population of 22,946 Icelanders. Cancer Epidemiol Biomarkers Prev. 6, 863–873 (1997). 9367058

[b12] Romero F. R., Romero A. W., Almeida R. M., Oliveira F. C. Jr. & Tambara Filho R. The significance of biological, environmental, and social risk factors for prostate cancer in a cohort study in Brazil. Int Braz J Urol. 38, 769–778 (2012). 23302414 10.1590/1677-553820133806769

[b13] Grundmark B. . The metabolic syndrome and the risk of prostate cancer under competing risks of death from other causes. Cancer Epidemiol Biomarkers Prev. 19, 2088–2096 (2010). 20647401 10.1158/1055-9965.EPI-10-0112 PMC2923431

[b14] Tande A. J., Platz E. A. & Folsom A. R. The metabolic syndrome is associated with reduced risk of prostate cancer. Am J Epidemiol. 164, 1094–1102 (2006). 16968859 10.1093/aje/kwj320

[b15] Stocks T., Hergens M. P., Englund A., Ye W. & Stattin P. Blood pressure, body size and prostate cancer risk in the Swedish Construction Workers cohort. Int J Cancer 127, 1660–1668 (2010). 20087861 10.1002/ijc.25171

[b16] Su Y. L., Chou C. L., Rau K. M. & Lee C. T. Asthma and Risk of Prostate Cancer: A Population-Based Case-Cohort Study in Taiwan. Medicine (Baltimore) 94, e1371 (2015). 26356691 10.1097/MD.0000000000001371 PMC4616655

[b17] Bhindi B. . Dissecting the association between metabolic syndrome and prostate cancer risk: analysis of a large clinical cohort. Eur Urol. 67, 64–70 (2015). 24568896 10.1016/j.eururo.2014.01.040

[b18] Wallner L. P. . The effects of metabolic conditions on prostate cancer incidence over 15 years of follow-up: results from the Olmsted County Study.BJU Int. 107, 929–935 (2011). 20880183 10.1111/j.1464-410X.2010.09703.x PMC3099535

[b19] Pelucchi C. . The metabolic syndrome and risk of prostate cancer in Italy. Ann Epidemiol. 21, 835–841 (2011). 21982487 10.1016/j.annepidem.2011.07.007

[b20] Martin R. M., Vatten L., Gunnell D. & Romundstad P. Blood pressure and risk of prostate cancer: Cohort Norway (CONOR). Cancer Causes Control 21, 463–472 (2010). 19949849 10.1007/s10552-009-9477-x

[b21] Inoue M. . Impact of metabolic factors on subsequent cancer risk: results from a large-scale population-based cohort study in Japan. Eur J Cancer Prev 18, 240–247 (2009). 19491612 10.1097/CEJ.0b013e3283240460

[b22] Beebe-Dimmer J. L. . Racial differences in risk of prostate cancer associated with metabolic syndrome. Urology 74, 185–190 (2009). 10.1016/j.urology.2009.03.013 PMC2704922 19428088

[b23] Tuohimaa P. . Interaction of factors related to the metabolic syndrome and vitamin D on risk of prostate cancer. Cancer Epidemiol Biomarkers Prev 16, 302–307 (2007). 10.1158/1055-9965.EPI-06-0777 17301263

[b24] Lund Haheim L., Wisloff T. F., Holme I. & Nafstad P. Metabolic syndrome predicts prostate cancer in a cohort of middle-aged Norwegian men followed for 27 years. Am J Epidemiol. 164, 769–774 (2006). 10.1093/aje/kwj284 16952929

[b25] Ronquist G. . Association between captopril, other antihypertensive drugs and risk of prostate cancer. Prostate 58, 50–56 (2004). 10.1002/pros.10294 14673952

[b26] Fitzpatrick A. L., Daling J. R., Furberg C. D., Kronmal R. A. & Weissfeld, J. L. Hypertension, heart rate, use of antihypertensives, and incident prostate cancer. Ann Epidemiol 11, 534–542 (2001). 10.1016/s1047-2797(01)00246-0 11709272

[b27] Rosengren A., Himmelmann A., Wilhelmsen L., Branehog I. & Wedel H. Hypertension and long-term cancer incidence and mortality among Swedish men. J Hypertens 16, 933–940 (1998). 10.1097/00004872-199816070-00006 9794733

[b28] Friedman G. D. Blood pressure and heart rate: no evidence for a positive association with prostate cancer. Ann Epidemiol. 7, 486–489 (1997). 10.1016/s1047-2797(97)00083-5 9349916

[b29] Esposito K. . Effect of metabolic syndrome and its components on prostate cancer risk: meta-analysis. J Endocrinol Invest 36, 132–139 (2013). 10.1007/BF03346748 23481613

[b30] Hadrava V., Tremblay J. & Hamet P. Abnormalities in growth characteristics of aortic smooth muscle cells in spontaneously hypertensive rats. Hypertension 13, 589–597 (1989). 10.1161/01.hyp.13.6.589 2786847

[b31] Mao Y., Xu X., Wang X., Zheng X. & Xie L. Is angiotensin-converting enzyme inhibitors/angiotensin receptor blockers therapy protective against prostate cancer? Oncotarget 7, 6765–6773 (2016). 10.18632/oncotarget.6837 PMC4872747 26760503

[b32] Häggström C. . Prospective study on metabolic factors and risk of prostate cancer. Cancer 15, 6199–6206 (2012). 10.1002/cncr.27677 23090855

[b33] Moher D., Liberati A., Tetzlaff J. & Altman D. G. Preferred reporting items for systematic reviews and meta-analyses: the PRISMA statement. Ann Intern Med. 151, 264–269, W264 (2009). 10.7326/0003-4819-151-4-200908180-00135 19622511

[b34] DerSimonian R. & Laird N. Meta-analysis in clinical trials. Control Clin Trials 7, 177–188 (1986). 10.1016/0197-2456(86)90046-2 3802833

[b35] Higgins J. P. & Thompson S. G. Quantifying heterogeneity in a meta-analysis. Stat Med. 21, 1539–1558 (2002). 10.1002/sim.1186 12111919

[b36] Begg C. B. & Mazumdar M. Operating characteristics of a rank correlation test for publication bias. Biometrics 50, 1088–1101 (1994). 7786990

[b37] Egger M., Davey Smith G., Schneider M. & Minder C. Bias in meta-analysis detected by a simple, graphical test. BMJ 315, 629–634 (1997). 10.1136/bmj.315.7109.629 PMC2127453 9310563

